# Elevated Monocyte-to-Lymphocyte and Platelet-to-Lymphocyte Ratios Are Associated with Disease Activity and Pain in Fibromyalgia: A Cross-Sectional Study

**DOI:** 10.3390/jcm15010155

**Published:** 2025-12-25

**Authors:** Meryem Kösehasanoğulları, Nilüfer Aygün Bilecik, Sıdıka Büyükvural Şen, Burhan Fatih Koçyiğit

**Affiliations:** Physical Medicine and Rehabilitation Clinic, Adana City Training and Research Hospital, Adana 01370, Turkey; drnilaygun@gmail.com (N.A.B.); sbuyukvuralsen@gmail.com (S.B.Ş.); bfk2701@hotmail.com (B.F.K.)

**Keywords:** fibromyalgia, inflammation, blood cell count, neutrophil-lymphocyte ratio, platelet-lymphocyte ratio, pain measurement

## Abstract

**Objective:** This study aimed to evaluate blood count-derived inflammatory indices—the neutrophil-to-lymphocyte ratio (NLR), platelet-to-lymphocyte ratio (PLR), monocyte-to-lymphocyte ratio (MLR), and the systemic immune-inflammation index (SII)—in patients with fibromyalgia and to explore their association with disease activity and pain severity. **Methods:** A cross-sectional study was conducted with 85 fibromyalgia patients and 84 age- and sex-matched healthy controls. Demographic, clinical, and laboratory data were recorded. Inflammatory indices were calculated from blood counts. Disease activity and functional status were assessed with the Fibromyalgia Impact Questionnaire (FIQ), Health Assessment Questionnaire (HAQ), and pain severity with the Visual Analog Scale (VAS). **Results:** Compared to controls, the fibromyalgia group had significantly higher BMI, PLR, MLR, and NLR (all *p* < 0.05), and lower lymphocyte levels. PLR and MLR moderately discriminated fibromyalgia (AUC = 0.623 and 0.661, respectively), suggesting limited diagnostic utility when used alone. MLR and BMI were independently associated with fibromyalgia in multivariate analysis. Disease duration showed significant positive correlations with PLR (r = 0.167), MLR (r = 0.228), FIQ (r = 0.773), HAQ (r = 0.589), and VAS at rest and movement (r = 0.584 and r = 0.601; all *p* < 0.05). PLR, MLR, and NLR were also positively correlated with VAS scores, while SII showed no significant associations. FIQ was strongly correlated with pain severity and HAQ with VAS during movement. **Conclusions:** Blood count-derived indices, particularly PLR and MLR, are elevated in fibromyalgia and are associated with disease duration, severity, and pain. Although PLR and MLR were higher in fibromyalgia patients, their discriminatory ability was limited and should be interpreted cautiously, indicating that their diagnostic specificity is low, as these ratios primarily reflect nonspecific inflammatory processes.

## 1. Introduction

Fibromyalgia is a complex chronic disorder characterized primarily by widespread musculoskeletal pain, persistent fatigue, sleep disturbances, and a range of cognitive and somatic symptoms [1]. Patients often experience a reduced quality of life, struggling with daily activities and emotional wellbeing due to the persistent and unpredictable nature of their symptoms [2]. The clinical presentation of fibromyalgia can vary greatly among individuals, sometimes making the diagnosis and management of the syndrome challenging for both patients and healthcare professionals [3].

The underlying mechanisms contributing to fibromyalgia remain incompletely understood, but a growing body of evidence suggests that immune system dysregulation and altered pain processing play crucial roles [4]. In recent years, inflammation has gained attention as a potential contributor to the development and persistence of fibromyalgia symptoms [5]. Although fibromyalgia is traditionally classified as a non-inflammatory condition, subtle changes in immune activity and low-grade inflammation have been observed in affected individuals, hinting at a more complex pathophysiology than previously thought [6]. Recent hypotheses propose that dysregulation within central neuro-immune pathways may contribute to a state of low-grade systemic inflammation in fibromyalgia, potentially mediated by glial cell activation and the release of pro-inflammatory cytokines [7].

Blood-based inflammatory markers, derived from routine complete blood counts, provide valuable insights into systemic immune activity [8]. Markers such as the neutrophil-lymphocyte ratio (NLR), monocyte-lymphocyte ratio (MLR), platelet-lymphocyte ratio (PLR) and systemic immune-inflammation index (SII) are easy to obtain, cost-effective, and reflect the balance between various immune cell populations [9]. Evaluating these markers in individuals with fibromyalgia may offer a better understanding of the immune alterations associated with the syndrome, as well as potential links to disease severity and symptom burden [10]. These indices have also been widely used as markers of systemic inflammation in other chronic pain and stress-related conditions, including migraine, chronic low back pain, and various rheumatic disorders, supporting their relevance as accessible indicators of low-grade inflammatory activation.

Recent conceptual models highlight that neurogenic inflammation and glial-cell dysregulation may play central roles in Fibromyalgia pathophysiology. Evidence from neuroimaging studies using PET with glial activation tracers (e.g., TSPO ligands) supports increased glial activity in pain-processing brain regions in FM patients [11]. Moreover, microglia, when activated, release pro-inflammatory cytokines—including IL-6, IL-8 and TNF-α—which contribute to neuronal sensitization, central sensitization and chronic pain [12]. On the other hand, peripheral immune dysregulation in FM is supported by meta-analytic data showing elevated circulating pro-inflammatory cytokines in patients vs. controls [13]. Consequently, systemic hematologic indices such as MLR and PLR may reflect not only peripheral immune cell alterations but also mirror a neuro-immune interface linking central glial activation with peripheral immune response in fibromyalgia.

The aim of this study is to assess blood-count-derived inflammatory markers in patients with fibromyalgia and to investigate their relationship with disease activity and pain levels. Although several studies have examined individual hematologic indices in fibromyalgia, few have evaluated multiple markers simultaneously, and none have assessed these indices in a treatment-naïve population or identified MLR as an independent factor in multivariate analysis. By incorporating these elements, the present study provides a more comprehensive evaluation of low-grade inflammation in fibromyalgia.

## 2. Material and Methods

### 2.1. Study Design

This study was designed as a prospective, cross-sectional study involving individuals who presented to the Physical Medicine and Rehabilitation outpatient clinic of Adana City Training and Research Hospital. Ethical approval for this study was obtained from the Scientific Research Ethics Committee of Adana City Training and Research Hospital (Protocol No: 174, Meeting No: 6, Approval Date: 10 October 2024). The study was conducted to evaluate inflammatory indices derived from blood count parameters in patients with fibromyalgia and to explore their clinical associations. All data were collected prospectively from the hospital’s electronic medical records and through direct patient evaluations between 11 October 2024, and 30 July 2025. Written informed consent was obtained from all participants prior to inclusion, in accordance with institutional and national ethical standards.

### 2.2. Participants

The study included 169 individuals, comprising 85 patients diagnosed with fibromyalgia according to the 2016 American College of Rheumatology (ACR) fibromyalgia diagnostic criteria and 84 age- and sex-matched healthy controls [14]. Patients were recruited consecutively from those who attended routine visits to the Physical Medicine and Rehabilitation outpatient clinic of Adana City Training and Research Hospital. Among the initially screened 92 patients with fibromyalgia, 7 (7.6%) were excluded due to incomplete clinical or laboratory data, leaving 85 patients eligible for analysis. The control group consisted of 84 healthy volunteers with no chronic diseases and complete laboratory data, recruited during routine outpatient health check-ups. Both groups were carefully matched in terms of demographic characteristics and basic health parameters.

### 2.3. Inclusion and Exclusion Criteria

The inclusion criteria for the study were: being between 18 and 65 years of age, having a diagnosis of fibromyalgia in the patient group, and having no chronic disease or major health problems in the control group, as well as possessing complete laboratory data in both groups. Individuals with thyroid disease, diabetes mellitus, hypertension, hyperlipidemia, other rheumatologic diseases, a history of malignancy or active cancer, pregnancy or the postpartum period were excluded from the study. Participants with systemic inflammatory diseases, active infections, recent (within the last 2–4 weeks) acute illnesses, smoking habits, or regular use of medications that could influence hematological parameters (e.g., NSAIDs, corticosteroids, antidepressants) were also excluded. These exclusions were applied to minimize the potential confounding effects of medications and comorbid conditions on inflammatory and hematologic indices. Smoking was specifically excluded because cigarette use is known to alter leukocyte distribution, elevate systemic inflammatory markers, and affect platelet activation, which could confound the interpretation of blood count–derived inflammatory indices.

### 2.4. Data Collection

Sociodemographic and clinical data of all participants were collected prospectively through face-to-face interviews and medical record documentation at the time of enrollment. The recorded demographic variables included age (years), sex, height (cm), body weight (kg), body mass index (BMI, kg/m^2^), marital status, educational level (primary school, secondary school, high school, university), occupation (homemaker, worker, civil servant, retired, self-employed), disease duration (years), and exercise habits. Exercise habits were categorized as follows: no regular exercise, exercising less than 3 days per week, and exercising at least 3 days per week for a minimum of 30 min per session. Low-to-moderate intensity exercise was defined as activities such as slow-to-brisk walking, gentle cycling, or comparable low-impact aerobic activities appropriate for individuals with chronic musculoskeletal pain. Clinical data included laboratory findings recorded at the time of admission and during previous visits. Laboratory parameters assessed were complete blood count (neutrophil, lymphocyte, monocyte, platelet, mean platelet volume [MPV, fL], platelet distribution width [PDW, fL]), serum biochemistry (total cholesterol, LDL, HDL, triglyceride; mg/dL), erythrocyte sedimentation rate (ESR, mm/h), and C-reactive protein (CRP, mg/L) levels. All blood samples were obtained from participants in the morning, between 08:00 and 10:00 a.m., after an overnight fast, to ensure standardization of laboratory measurements. The menstrual cycle phase was not controlled for in female participants, and therefore cycle-related variability in hematologic parameters cannot be excluded. The control group consisted of individuals attending the hospital for routine annual health check-ups, which included general physical examination, basic laboratory testing, and cardiovascular risk screening, and who did not report any symptoms or known chronic medical conditions.

### 2.5. Assessment Tools and Calculations

In all participants, pain severity, disease activity, and functional health status were assessed using various scales. Pain levels were determined separately at rest and during movement using a 10 cm horizontal Visual Analog Scale (VAS) scored from 0 (no pain) to 10 (worst imaginable pain) [15]. Disease activity was evaluated with the Fibromyalgia Impact Questionnaire (FIQ), which consists of 10 items assessing physical function, work status, depression, anxiety, sleep, pain, stiffness, fatigue, and overall health in patients; higher scores indicate greater disease severity [16]. Functional health status was assessed using the Health Assessment Questionnaire (HAQ), a validated and reliable instrument that evaluates daily living activities across eight domains and 20 items, scored between 0 and 3 [17].

The inflammation indices derived from blood count parameters were calculated using the following formulas:

- Neutrophil-to-Lymphocyte Ratio (NLR): NLR = Neutrophil/Lymphocyte
- Platelet-to-Lymphocyte Ratio (PLR): PLR = Platelet/Lymphocyte
- Monocyte-to-Lymphocyte Ratio (MLR): MLR = Monocyte/Lymphocyte
- Systemic Immune-Inflammation Index (SII): SII = (Neutrophil × Platelet)/Lymphocyte

### 2.6. Statistical Analysis

Statistical analyses were performed using SPSS software (version 27.0; IBM Corp., Armonk, NY, USA). Continuous variables were expressed as mean ± standard deviation (SD), while categorical variables were presented as numbers and percentages. The normality of distribution was assessed using the Shapiro–Wilk test. Additionally, variables that showed highly skewed distributions (e.g., NLR, PLR, SII) were analyzed using non-parametric tests. Normality was assessed with the Shapiro–Wilk test and by visual inspection of histograms and Q–Q plots. For comparisons between two independent groups, the independent samples t-test was used for normally distributed variables, and the Mann–Whitney U test was applied when the normality assumption was not met. Categorical variables were compared using the Chi-square test or Fisher’s exact test where appropriate. Correlations between continuous variables were assessed using Pearson or Spearman correlation coefficients, depending on the distribution. Receiver operating characteristic (ROC) curve analysis was performed to evaluate the discriminatory ability of inflammatory indices, and the area under the curve (AUC) with 95% confidence intervals was calculated. Multivariate logistic regression analysis (stepwise method) was used to determine independent factors associated with fibromyalgia syndrome. A *p* value < 0.05 was considered statistically significant. The sample size was calculated using the G*Power 3.1.9.7 software (Heinrich-Heine-University, Düsseldorf, Germany). Based on previous studies evaluating inflammatory indices in fibromyalgia and assuming a medium effect size (Cohen’s d = 0.5), a power of 0.80, and a two-tailed α error of 0.05, the minimum required sample size was determined as 64 participants per group. To account for potential data loss, 85 fibromyalgia patients and 84 controls were included, ensuring adequate statistical power for the analyses. Outliers were checked by visual inspection of scatter plots and standardized residuals; none exerted undue influence on correlation estimates. Additionally, partial correlations adjusting for age, sex, BMI, and physical activity were conducted to assess the robustness of associations.

## 3. Results

The mean ages were 46.60 ± 8.61 and 45.40 ± 9.71 years in the fibromyalgia and control groups, respectively, with no significant difference between the groups (*p* = 0.288). The mean height was significantly lower in the fibromyalgia group (161.10 ± 4.52 cm) compared to the control group (163.40 ± 4.52 cm; *p* < 0.001). Body weight was slightly higher in the fibromyalgia group (66.00 ± 10.33 kg) than in the control group (63.40 ± 8.31 kg), although this difference did not reach statistical significance (*p* = 0.060). BMI values were comparable between the groups (25.60 ± 4.61 vs. 24.80 ± 3.72 kg/m^2^; *p* = 0.095) (Table 1).

There were no significant differences between the groups in terms of marital status, educational level, or occupation (*p* = 0.252, *p* = 0.361, and *p* = 0.348, respectively). Although the proportion of individuals who did not exercise was higher in the fibromyalgia group (50.6%) compared to the control group (41.7%), this difference did not reach statistical significance (*p* = 0.061) (Table 2).

In the fibromyalgia group, lymphocyte levels were significantly lower (2.13 ± 0.72 vs. 2.60 ± 0.83 × 10^9^/L; *p* < 0.001), whereas PDW (13.44 ± 2.94 vs. 14.33 ± 2.60 fL; *p* = 0.032), PLR (159.80 ± 123.41 vs. 124.32 ± 48.81; *p* = 0.007), MLR (0.31 ± 0.22 vs. 0.21 ± 0.12; *p* < 0.001), and NLR (2.52 ± 2.72 vs. 1.93 ± 0.73; *p* = 0.043) were significantly higher compared to the control group. SII was also higher in the fibromyalgia group (696.40 ± 715.90 vs. 539.20 ± 231.40), and this difference reached statistical significance (*p* = 0.047). No significant differences were observed for the remaining laboratory parameters. Clinically, FIQ and HAQ scores, as well as VAS values at rest and during movement, were markedly higher in the fibromyalgia group than in the control group (all *p* < 0.001) (Table 3).

There was a significant positive correlation between disease duration and both PLR (r = 0.167, *p* = 0.029) and MLR (r = 0.228, *p* = 0.003). In addition, disease duration showed strong positive correlations with FIQ (r = 0.773, *p* < 0.001), HAQ (r = 0.589, *p* < 0.001), VAS at rest (r = 0.584, *p* < 0.001), and VAS during movement (r = 0.601, *p* < 0.001). PLR and MLR were also positively correlated with VAS (resting and movement), with significant but weaker correlations (PLR: r = 0.163, *p* = 0.034 for resting, r = 0.180, *p* = 0.019 for movement; MLR: r = 0.256, *p* = 0.001 for resting, r = 0.239, *p* = 0.002 for movement). NLR was modestly correlated with VAS scores (NLR: r = 0.162, *p* = 0.035 for resting, r = 0.152, *p* = 0.048 for movement). SII did not show significant correlations with disease duration or VAS scores. FIQ score showed strong positive correlations with both VAS at rest (r = 0.531, *p* < 0.001) and VAS during movement (r = 0.511, *p* < 0.001), while HAQ was moderately correlated with VAS during movement (r = 0.315, *p* = 0.003). No significant associations were observed between SII and other parameters (Table 4).

**Figure 1:**
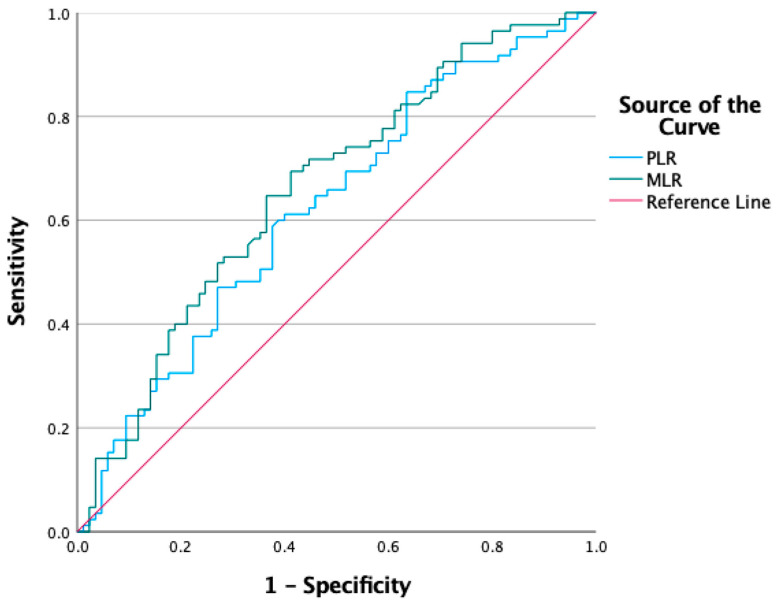
ROC Curves of PLR and MLR for Discriminating Fibromyalgia Syndrome.

For PLR, the AUC was 0.623, with a cut-off value of 120.5, sensitivity of 60%, and specificity of 62%, and this result was statistically significant (*p* = 0.006; 95% CI: 0.539–0.707). For MLR, the AUC was 0.661, with a cut-off value of 0.24, sensitivity of 70%, and specificity of 60%, and this finding was also statistically significant (*p* < 0.001; 95% CI: 0.580–0.743) (Table 5) (Figure 1).

In the multivariate logistic regression analysis of factors associated with fibromyalgia syndrome, both MLR (B = 5.358, *p* = 0.002, Exp(B) = 212.3; 95% CI: 7.1–636.5) and body mass index (BMI) (B = 0.101, *p* = 0.018, Exp(B) = 1.106; 95% CI: 1.017–1.202) were found to be significantly associated (Table 6). ROC analyses were also performed for NLR and SII; however, their AUC values were below 0.60 and did not reach statistical significance. Therefore, these indices were not included in the ROC figure due to their limited discriminatory performance.

## 4. Discussion

In our study, we found that patients with fibromyalgia syndrome had a higher body mass index compared to the control group, although this difference was not statistically significant. Certain blood count–based inflammatory markers, including PLR, MLR, and NLR, were significantly elevated in the fibromyalgia group, while lymphocyte levels were lower. Furthermore, when evaluating the clinical status of the patients, disease severity, functional limitation, and pain levels were markedly increased in the fibromyalgia group. Physical activity levels, on the other hand, were lower in patients with fibromyalgia. These findings indicate that fibromyalgia syndrome may affect both the inflammatory response and quality of life in a multidimensional manner. Our results support the role of immune system–related parameters in the pathophysiology of fibromyalgia and highlight the need for further research into the relationship between clinical features of the disease and inflammatory markers.

Almirall et al. reported higher NLR values in fibromyalgia patients compared with controls and noted a further increase among those with greater disease severity [18]. Consistent with these findings, our study also demonstrated significantly elevated NLR, PLR, and MLR levels in the fibromyalgia group, supporting the notion that multiple hematologic markers may be altered in this condition. Fibromyalgia is frequently accompanied by symptoms with inflammatory features, yet whether it constitutes an inflammatory disorder remains debated. Classical markers such as ESR and CRP show inconsistent results [19,20,21,22,23]; for example, a large cohort identified elevated CRP levels that diminished after adjustment for BMI and comorbidities [23]. Ege et al. found increased leukocyte subsets and lower PDW but no differences in NLR or PLR [24], whereas our study identified significant elevations in NLR, PLR, and MLR along with reduced lymphocytes, highlighting variability across patient populations. Although Ege et al. reported higher MPV values, our results did not replicate this. Collectively, these discrepancies—particularly regarding NLR and PLR—underscore the heterogeneity of hematologic responses in fibromyalgia and the need for further investigation. An additional finding that merits emphasis is the significantly lower lymphocyte count in the fibromyalgia group. This lymphopenia may contribute substantially to the elevation of lymphocyte-derived indices such as NLR, PLR, and MLR, suggesting that altered lymphocyte dynamics could be a central component of the inflammatory profile observed in fibromyalgia.

Another plausible pathogenic mechanism involves chronic stress–related dysregulation of the autonomic nervous system and the hypothalamic–pituitary–adrenal (HPA) axis, both of which are frequently impaired in fibromyalgia. Chronic stress can suppress lymphocyte proliferation and promote lymphocyte redistribution, contributing to lymphopenia, while simultaneously enhancing the mobilization of monocytes and the activation of platelets through catecholamine-mediated pathways. These stress-driven alterations may partly explain the elevated MLR and PLR values observed in our study and further support the concept of a systemic neuroendocrine–immune imbalance in fibromyalgia, as described in previous work linking sympathetic overactivity and HPA axis dysregulation to immune shifts in FM [25].

Recent evidence supports the involvement of peripheral immune cells and neuroimmune signaling in fibromyalgia. In particular, studies have demonstrated elevated expression of pro-inflammatory cytokines such as IL-6, IL-8, and TNF-α in monocytes from fibromyalgia patients, suggesting monocyte activation may contribute to systemic and central sensitization processes [5,13,26]. Moreover, platelets are increasingly recognized not only for hemostatic functions but also for their capacity to modulate nociceptive pathways via release of serotonin, pro-inflammatory mediators, and platelet-derived cytokines. Thus, the elevated MLR and PLR values observed in our study may reflect a neuro-immune interface in which peripheral monocyte and platelet activation contribute to altered pain processing and chronicity in fibromyalgia. This framework provides a biological rationale for the association between hematologic indices and clinical features of fibromyalgia, beyond simple hematologic variation.

Jayakrishnan et al. reported mean NLR, PLR, and MPV values of 1.92 ± 1.26, 119.48 ± 76.91, and 8.94 ± 1.25 in 266 fibromyalgia patients, noting no association between NLR or PLR and disease severity, while MPV showed a mild inverse correlation with FIQR scores [27]. In contrast, our study found significantly higher NLR, PLR, and MLR values compared with controls, with NLR demonstrating an association with disease activity, although MPV did not differ between groups. Recent studies have similarly suggested that NLR and PDW may serve as potential inflammatory biomarkers in fibromyalgia [20], and Al-Nimer et al. reported a relationship between NLR and disease activity [28]. Additional evidence from nosiplastic pain conditions—including migraine—indicates the presence of low-grade inflammation even during pain-free periods [29,30]. Raikan et al. further demonstrated elevations in several hematologic indices such as NLR, ELR, EC, PDW, SII, and SIRI, and proposed an NLR cutoff of 1.62, albeit with modest diagnostic performance [31]. Collectively, these findings indicate that blood count–derived inflammatory markers—including those evaluated in our study—may contribute supportive information in fibromyalgia assessment, although their limited specificity restricts their use as independent diagnostic tools. Given their nonspecific nature, these ratios may also be influenced by chronic stress, obesity, depressive symptoms, or other comorbidities frequently observed in individuals with fibromyalgia. Therefore, the elevations observed in our cohort likely reflect broader systemic low-grade inflammation rather than a fibromyalgia-specific inflammatory signature.

Although these hematologic ratios are attractive due to their simplicity, low cost, and clinical accessibility, their limited specificity remains a major challenge. Other proposed biomarkers in fibromyalgia, such as serum brain-derived neurotrophic factor (BDNF) levels or cerebrospinal fluid cytokine profiles, may offer greater pathophysiological specificity but require specialized assays or invasive procedures, making them impractical for routine clinical use. In this context, MLR and PLR may be best regarded as supportive rather than diagnostic indicators of low-grade systemic inflammation.

In the study by Tertemiz and Tepe, although the neutrophil-to-lymphocyte ratio (NLR) was found to be higher in patients with fibromyalgia compared to the control group (1.94 ± 0.8 vs. 1.54 ± 0.6), this difference was not statistically significant (*p* > 0.05) [32]. Additionally, the study aimed to objectively assess perceptual functions using the two-point discrimination test (STD), and the STD duration was shown to be significantly longer in patients with fibromyalgia. In our study, not only was the NLR value found to be significantly higher in the fibromyalgia group, but other hemogram parameters such as PLR and MLR were also shown to be significantly increased. While the relationship between NLR and pain or disease severity was not evaluated in the study by Tertemiz and Tepe, our results demonstrated a positive association between NLR and disease activity. Furthermore, whereas Tertemiz and Tepe suggested that the two-point discrimination test may be used for objective diagnosis, our study highlighted the potential utility of hematological markers in the assessment of diagnosis and disease severity.

Recent studies evaluating hematologic inflammatory biomarkers in fibromyalgia have yielded mixed findings. Koca et al. reported higher NLR, PLR, MLR, SII, and SIRI but no differences in MPV or PDW [33], whereas El-Sawy et al. found elevated ESR, CRP, NLR, and MPV, with PDW unchanged [34]. Varim et al. observed increased NLR and PLR but lower LMR, further illustrating variability across studies [35]. Jayakrishnan et al., in a larger cohort, reported mean NLR, PLR, and MPV values of 1.92 ± 1.26, 119.48 ± 76.91, and 8.94 ± 1.25, respectively, noting only a weak inverse correlation between MPV and symptom severity, while NLR and PLR were not associated with disease activity [27]. These inconsistencies likely reflect differences in sample size, patient characteristics, methodological adjustments, and analytical approaches. Collectively, the heterogeneity suggests that although hematologic indices may indicate low-grade inflammation in fibromyalgia, none can yet be regarded as definitive diagnostic or prognostic markers without further validation.

This study has several limitations that should be considered when interpreting the results. First, it was conducted at a single tertiary care center, which reduces the external validity and limits the generalizability of the findings. Second, due to its cross-sectional design, the study can only demonstrate associations rather than causality between inflammatory indices and fibromyalgia syndrome. Therefore, it remains uncertain whether the observed alterations in inflammatory indices represent primary mechanisms contributing to fibromyalgia or secondary responses to chronic pain, psychological stress, and long-standing maladaptation. In addition, since participants were recruited from a hospital outpatient clinic, potential selection bias cannot be excluded. Finally, some clinical variables—including exercise habits and pain severity—were based on self-reported data, which may be subject to recall or reporting bias. Although matching was applied for age and sex, residual confounding by demographic or lifestyle factors cannot be fully excluded. However, excluding patients who were regularly using antidepressants, NSAIDs, or corticosteroids may have limited the external generalizability of the findings, as such medications are commonly used in the broader fibromyalgia population.

Despite these limitations, this study has several strengths. First, it included a treatment-naive fibromyalgia population, minimizing the potential confounding effects of pharmacologic therapy on hematological parameters. Second, the sample size was statistically adequate, as determined by a priori power analysis. Third, the inflammatory indices evaluated in this study are simple, inexpensive, and routinely available markers, which increase the clinical applicability of the findings. Finally, to our knowledge, this is one of the few studies to identify the monocyte-to-lymphocyte ratio (MLR) as an independent factor associated with fibromyalgia, providing novel insight into the role of low-grade inflammation in the disease.

## 5. Conclusions

In conclusion, this study demonstrated that inflammation indices derived from routine blood count parameters—particularly the MLR and PLR—were significantly higher in patients with fibromyalgia syndrome compared to healthy controls. Multivariate analysis revealed that both increased MLR and higher BMI were independently associated with fibromyalgia. Although PLR and MLR showed positive correlations with disease duration, the *p*-values were marginal and may be sensitive to multiple comparison considerations; therefore, these findings should be interpreted cautiously. The novel aspect of this study lies in identifying the monocyte-to-lymphocyte ratio (MLR) as an independent inflammatory marker associated with fibromyalgia, which has not been clearly demonstrated in previous research. Moreover, our findings highlight that both PLR and MLR are not only elevated but also correlated with clinical parameters such as pain and disease duration, providing new insights into the role of low-grade inflammation in fibromyalgia. MLR and PLR may reflect mild immune alterations in fibromyalgia; however, their limited effect sizes and AUC values indicate that they are not suitable for clinical evaluation. Although these indices show modest absolute differences, they may still have potential value as supportive markers for monitoring longitudinal changes in systemic inflammation. Future studies are needed to determine whether dynamic shifts in MLR, PLR, or NLR could assist in tracking treatment response or disease fluctuations. However, further large-scale, multicenter, and longitudinal studies are needed to confirm these results and to explore whether these hematologic indices can serve as supportive tools for disease assessment and treatment monitoring in fibromyalgia.

## Figures and Tables

**Table 1:** Comparison of Basic Demographic Characteristics Between Fibromyalgia and Control Groups.

	Fibromyalgia (*n* = 85)	Control (*n* = 84)	
	Mean ± S.D.	Mean ± S.D.	*p* Value
**Age (years)**	46.6 ± 8.61	45.4 ± 9.71	0.288
**Height (cm)**	161.1 ± 4.52	163.4 ± 4.52	**<0.001**
**Body weight (kg)**	66.0 ± 10.33	63.4 ± 8.31	0.060
**BMI (kg/m^2^)**	25.6 ± 4.61	24.8 ± 3.72	0.095

Data are presented as mean ± standard deviation. Abbreviations: BMI, body mass index; S.D., standard deviation. Statistically significant *p* values are shown in bold.

**Table 2:** Comparison of Sociodemographic Characteristics Between Fibromyalgia and Control Groups.

		Fibromyalgia (*n* = 85)	Control (*n* = 84)	
		Count (%)	Count (%)	*p* Value
**Marital status**	Single	6 (7.1)	11 (13.9)	0.252
	Married	79 (92.9)	73 (86.1)	
**Educational level**	Elementary	34 (40.0)	24 (28.2)	0.361
	Secondary School	16 (18.8)	15 (17.6)	
	High school	24 (28.2)	29 (34.3)	
	University	11 (12.9)	16 (18.8)	
**Occupation**	Housewife	55 (64.7)	43 (50.6)	0.348
	Worker	7 (8.2)	9 (10.6)	
	Civil Servant	13 (15.3)	19 (22.5)	
	Retired	4 (4.7)	8 (9.4)	
	Self-Employed	6 (7.1)	5 (5.9)	
**Exercise status**	No	43 (50.6)	35 (41.7)	0.061
	<3 Weeks	19 (22.4)	14 (16.6)	
	≥3 Weeks	23 (27.1)	35 (41.7)	

Data are presented as number (percentage). Statistically significant *p* values are shown in bold.

**Table 3:** Comparison of Laboratory and Clinical Parameters Between Fibromyalgia and Control Groups.

	Fibromyalgia (*n* = 85)	Control (*n* = 84)	
	Mean ± S.D.	Mean ± S.D.	*p* Value
**Neutrophil (10^9^/L)**	4.21 ± 1.40	4.51 ± 1.32	0.091
**Lymphocyte (10^9^/L)**	2.13 ± 0.72	2.6 ± 0.83	**<0.001**
**Monocyte (10^9^/L)**	0.53 ± 0.21	0.52 ± 0.20	0.635
**Platelet (10^9^/L)**	279.52 ± 55.94	291.62 ± 57.21	0.212
**MPV (fL)**	10.01 ± 1.23	10.01 ± 1.23	0.954
**PDW (fL)**	13.44 ± 2.94	14.33 ± 2.60	**0.032**
**Total cholesterol (mg/dL)**	205.82 ± 40.41	199.74 ± 37.81	0.340
**LDL (mg/dL)**	135.61 ± 31.42	130.62 ± 30.32	0.352
**HDL (mg/dL)**	55.06 ± 11.60	53.93 ± 10.50	0.444
**Triglyceride (mg/dL)**	135.22 ± 58.41	126.10 ± 54.10	0.332
**ESR (mm/h)**	10.33 ± 6.12	9.52 ± 5.11	0.464
**CRP (mg/L)**	2.91 ± 1.44	2.51 ± 1.22	0.117
**NLR**	1.8 (1.3–2.2)	1.9 (1.4–2.6)	**0.043**
**PLR**	114.7 (96.5–148.7)	133.5 (104.7–166.9)	**0.007**
**MLR**	0.20 (0.20–0.30)	0.30 (0.20–0.40)	**<0.001**
**SII**	515.2 (395.4–638.6)	497.3 (363.1–779.0)	**0.047**
**FIQ Score**	58.1 ± 10.7	10.9 ± 5.7	**<0.001**
**HAQ Score**	1.1 ± 0.5	0.1 ± 0.1	**<0.001**
**VAS (resting)**	4.9 ± 1.7	1.6 ± 1.1	**<0.001**
**VAS (movement)**	7.7 ± 1.0	2.2 ± 1.0	**<0.001**

Data are presented as mean ± standard deviation and median (interquartile range). Abbreviations: MPV, mean platelet volume; PDW, platelet distribution width; ESR, erythrocyte sedimentation rate; CRP, C-reactive protein; NLR, neutrophil-to-lymphocyte ratio; PLR, platelet-to-lymphocyte ratio; MLR, monocyte-to-lymphocyte ratio; SII, systemic immune-inflammation index; FIQ, Fibromyalgia Impact Questionnaire; HAQ, Health Assessment Questionnaire; VAS, visual analog scale; LDL, low-density lipoprotein; HDL, high-density lipoprotein. Statistically significant *p* values are shown in bold.

**Table 4:** Pearson Correlation Between Disease Duration and Clinical/Laboratory Parameters in Fibromyalgia Group.

		Disease Duration (Years)	VAS (Resting)	VAS (Movement)
**NLR**	Pearson Correlation	0.119	0.162	0.152
	*p* value	0.123	**0.035**	**0.048**
**PLR**	Pearson Correlation	0.167	0.163	0.180
	*p* value	**0.029**	**0.034**	**0.019**
**MLR**	Pearson Correlation	0.228	0.256	0.239
	*p* value	**0.003**	**0.001**	**0.002**
**SII**	Pearson Correlation	0.118	0.146	0.139
	*p* value	0.124	0.057	0.072
**FIQ Score**	Pearson Correlation	0.773	0.531	0.511
	*p* value	**<0.001**	**<0.001**	**<0.001**
**HAQ Score**	Pearson Correlation	0.589	0.181	0.315
	*p* value	**<0.001**	0.098	**0.003**
**VAS (resting)**	Pearson Correlation	0.584	-	-
	*p* value	**<0.001**	**-**	**-**
**VAS (movement)**	Pearson Correlation	0.601	-	-
	*p* value	**<0.001**	**-**	**-**

Abbreviations: NLR, neutrophil-to-lymphocyte ratio; PLR, platelet-to-lymphocyte ratio; MLR, monocyte-to-lymphocyte ratio; SII, systemic immune-inflammation index; FIQ, Fibromyalgia Impact Questionnaire; HAQ, Health Assessment Questionnaire; VAS, visual analog scale. Statistically significant *p* values are shown in bold.

**Table 5:** ROC Analysis Results of PLR and MLR for Discriminating Fibromyalgia Syndrome.

	AUC	Cut-Off	Sensitivity	Specificity	*p* Value	Asymp 95% CI	
						Lower Bound	Upper Bound
**PLR**	0.623	120.5	%60	%62	**0.006**	0.539	0.707
**MLR**	0.661	0.24	%70	%60	**<0.001**	0.580	0.743

AUC, area under the curve; PLR, platelet-to-lymphocyte ratio; MLR, monocyte-to-lymphocyte ratio; CI, confidence interval. Statistically significant *p* values are shown in bold.

**Table 6:** Stepwise Multivariate Logistic Regression Analysis of Factors Associated with Fibromyalgia Syndrome.

					95% CI for EXP(B)	
	B	S.E.	*p* Value	Exp(B)	Lower	Upper
**MLR**	5.358	1.735	**0.002**	212.3	7.1	636.5
**BMI (kg/m^2^)**	0.101	0.043	**0.018**	1.106	1.017	1.202
**Constant**	−1.127	1.141	0.323	0.324		

Abbreviations: MLR, monocyte-to-lymphocyte ratio; BMI, body mass index; Exp(B), exponentiated coefficient (odds ratio); CI, confidence interval. Statistically significant *p* values are shown in bold.

## Data Availability

The data that support the findings of this study are available from the corresponding author upon reasonable request.
